# Reactive Sterol Electrophiles: Mechanisms of Formation and Reactions with Proteins and Amino Acid Nucleophiles^†^

**DOI:** 10.3390/chemistry2020025

**Published:** 2020-05-06

**Authors:** Ned A. Porter, Libin Xu, Derek A. Pratt

**Affiliations:** 1 Department of Chemistry and Vanderbilt Institute of Chemical Biology, Vanderbilt University, Nashville, TN 37235, USA; 2 Department of Medicinal Chemistry, University of Washington, Seattle, WA 98195, USA;; 3 Department of Chemistry and Biomolecular Sciences, University of Ottawa, 10 Marie Curie Pvt., Ottawa, ON K1N 6N5, Ontario, Canada;

**Keywords:** peroxidation, free radical, sterol, cholesterol, lipid electrophiles

## Abstract

Radical-mediated lipid oxidation and the formation of lipid hydroperoxides has been a focal point in the investigation of a number of human pathologies. Lipid peroxidation has long been linked to the inflammatory response and more recently, has been identified as the central tenet of the oxidative cell death mechanism known as ferroptosis. The formation of lipid electrophile-protein adducts has been associated with many of the disorders that involve perturbations of the cellular redox status, but the identities of adducted proteins and the effects of adduction on protein function are mostly unknown. Both cholesterol and 7-dehydrocholesterol (7-DHC), which is the immediate biosynthetic precursor to cholesterol, are oxidizable by species such as ozone and oxygen-centered free radicals. Product mixtures from radical chain processes are particularly complex, with recent studies having expanded the sets of electrophilic compounds formed. Here, we describe recent developments related to the formation of sterol-derived electrophiles and the adduction of these electrophiles to proteins. A framework for understanding sterol peroxidation mechanisms, which has significantly advanced in recent years, as well as the methods for the study of sterol electrophile-protein adduction, are presented in this review.

## 1. Introduction

Unsaturated lipids are prone to undergoing reactions with oxidizing species, such as oxygen-centered free radicals, singlet molecular oxygen, and ozone. The high abundance of these vulnerable lipids in humans is associated with pathologies that result from exposure to such reactive oxidants. Ozone, for example, is the most widespread air pollutant found in the U.S. and contributes to a growing variety of health problems, all of which potentially increase the risk of premature death [1,2]. Reactive oxygen species (ROS), such as alkoxyl and peroxyl free radicals, are generally linked to oxidative stress, which has been associated with many human disorders. Indeed, ROS and free radical lipid peroxidation has been invoked as a cause or consequence in diseases such as asthma [3], cardiovascular disease [4–6], diabetes [5,7,8], Alzheimer’s [9–11], Parkinson’s [12,13], cancer [14,15], and macular degeneration [16]. More recently, a form of regulated necrosis associated with the accumulation of lipid hydroperoxides has been characterized, which may link specific oxidative events and tissue dysfunction in these pathological contexts. This process, coined ferroptosis, which may also serve as a vulnerability that may be exploited for cancer treatment, implicates a labile iron pool and lipid hydroperoxides as causal agents and a glutathione-dependent enzyme (GPX4), radical trapping antioxidants (RTAs), and iron chelating compounds as protective agents [17–19].

Reactive electrophiles have been suggested to be products of lipid peroxidation since the 1940s, when thiobarbituric acid (TBA) was shown to give a characteristic red-orange color with animal tissue that had been exposed to air [20]. The colored lipid-derived species was subsequently identified as a 2:1 complex of TBA with malondialdehyde (MDA) [21,22]—an electrophilic byproduct of the free radical peroxidation of polyunsaturated fatty acids (PUFAs) and esters with three or more double bonds. While a number of methods have been developed over the decades to assay the overall levels of peroxidation and identify specific products of lipid oxidation, the TBARS assay (TBA ‘reactive species’) is still used to give a semi-quantitative measure of MDA levels.

At the time that reactive lipid-derived electrophiles like MDA were being associated with lipid oxidation reactions in animal tissues, the chemistry of free radical chain reactions responsible for their formation was also drawing significant attention. While early work on the mechanism of autoxidation was principally centered on the degradation of commercially important hydrocarbons, lipid peroxidation drew increased interest during the latter half of the 20th century, with efforts to describe mechanisms for the autoxidation of polyunsaturated fatty acids (PUFAs) [23–30] and sterols [27,31–42] attracting interest that has continued to this day.

The chemistry and biology of PUFA-derived electrophiles has drawn attention since MDA was shown to be present in cells, fluids, and tissues under conditions of oxidative stress. Mechanisms for its formation were proposed [43,44], and routes were suggested for the formation of other fatty acid-derived electrophiles, such as the cytotoxins 4-hydroxy-2-nonenal (4-HNE) and 5-oxo-2-nonenal (4-ONE) [45–49]. Another set of reactive electrophiles with a core 4-oxo-pentanal structure, the isolevuglandins (IsoLGs), were also found in cells, tissues, and fluids undergoing oxidative stress. Mechanisms for the formation of IsoLGs centered on the decomposition of endoperoxide intermediates formed in the peroxidation of arachidonate esters [50–53]. In fact, the same unstable endoperoxide intermediate serves as a precursor to both MDA and the IsoLGs.

In the decades following the identification of MDA as a byproduct of lipid peroxidation, the formation and repair of DNA adducts caused by MDA was the subject of extensive investigation [14]. Adducts of 4-HNE and 4-ONE with DNA have also drawn interest, leading to suggestions that small-molecule electrophile adduction to nucleic acids may be a major cause of cancers linked to lifestyle and dietary factors [15].

Reactions between PUFA-derived electrophiles and protein nucleophiles have also been studied in detail. MDA-promoted crosslinking of proteins has been linked to the formation of the fluorescent age-related pigment lipofuscin [54], and the protein adduction of 4-HNE, 4-ONE, and IsoLGs has been the subject of extensive investigation and several excellent reviews [55–57]. MDA and IsoLG adduction to proteins generally involves initial reversible imine formation, which is generally followed by molecular rearrangement to give stable end-products. The α,β-unsaturated aldehydes (4-HNE and 4-ONE) usually undergo Michael addition with cysteines, histidines, and lysines.

Free radical-, singlet oxygen-, and ozone-promoted cholesterol oxidation also leads to several reactive compounds [31–38], but the reactions of these sterol-derived electrophiles with biological nucleophiles has drawn relatively little attention compared to the many studies on PUFA-derived compounds such as MDA and 4-HNE. Cholesterol levels are particularly high in the brain and central nervous system and neurons are among the most vulnerable cells to reactive oxygen species (ROS), due to their elevated metabolic activity, highly unsaturated neuronal lipid composition (such as docosahexaenoic acid, arachidonic acid, and cholesterol), high level of transition metals, and modest antioxidant defense systems [58,59]. This background, along with the suggestion that reactive oxysterols may promote protein aggregation [10,11,60], has led to an increase in interest in the chemistry and biology of cholesterol-derived electrophiles.

**Figure 1.**
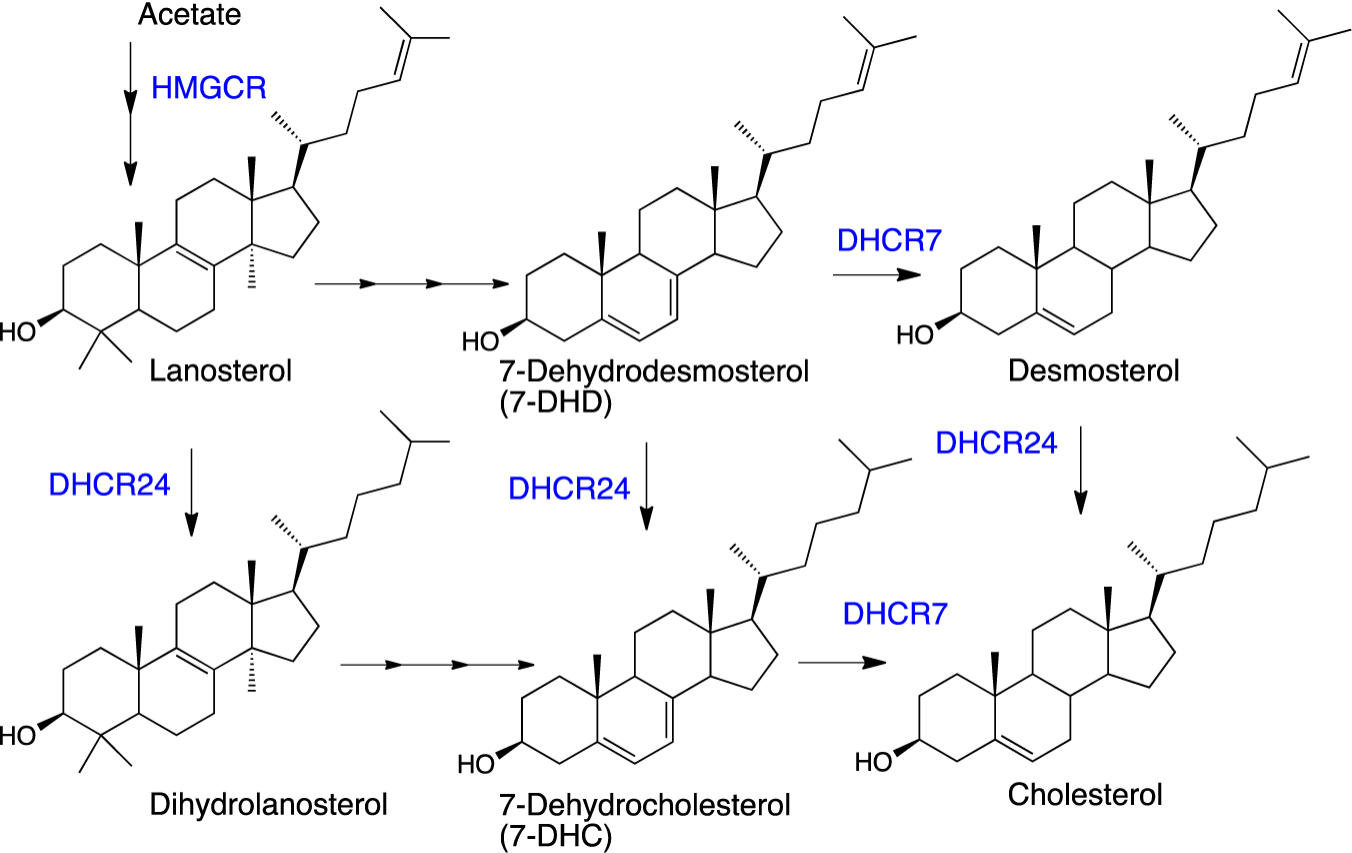
Selected cholesterol biosynthetic precursors and enzymes (in blue) that promote the transformations shown.

Recent discoveries have stimulated interest in post-lanosterol sterols other than cholesterol. One of the immediate biosynthetic precursors to cholesterol, 7-dehydrocholesterol (7-DHC, see Figure 1), is highly vulnerable to free radical chain oxidation [61] and reactive electrophiles are major products of its peroxidation [62,63]. Elevated levels of 7-DHC are found in tissues and fluids of patients with the genetic disorder Smith-Lemli-Opitz syndrome (SLOS) [64], which is caused by mutations in the gene encoding 7-dehydrocholesterol reductase (DHCR7)—the enzyme that converts 7-DHC to cholesterol [65]. The high levels of 7-DHC found in SLOS tissues, the proclivity of this sterol to participate in radical chain oxidation reactions, and the formation of highly reactive sterol electrophiles in the process have led to recent suggestions that SLOS is a disorder driven by lipid peroxidation [63,66–69].

The increased interest in sterol-derived electrophiles, their reactions with biologically important nucleophiles, and the link between these reactive species and human disorders, including neurodegenerative disease, were the principal stimuli for undertaking this review. In this work, we describe recent advances in the chemistry leading to sterol-derived electrophiles and the reaction of these species with proteins, peptides, and amino acids. An introduction to the chemical tools developed to date to study electrophile-protein adduction and a discussion of what insights these have provided are a part of this contribution, as are the potential biological consequences of the formation and transformations of these species.

## 2. Primary Reactions

**Figure 2.**
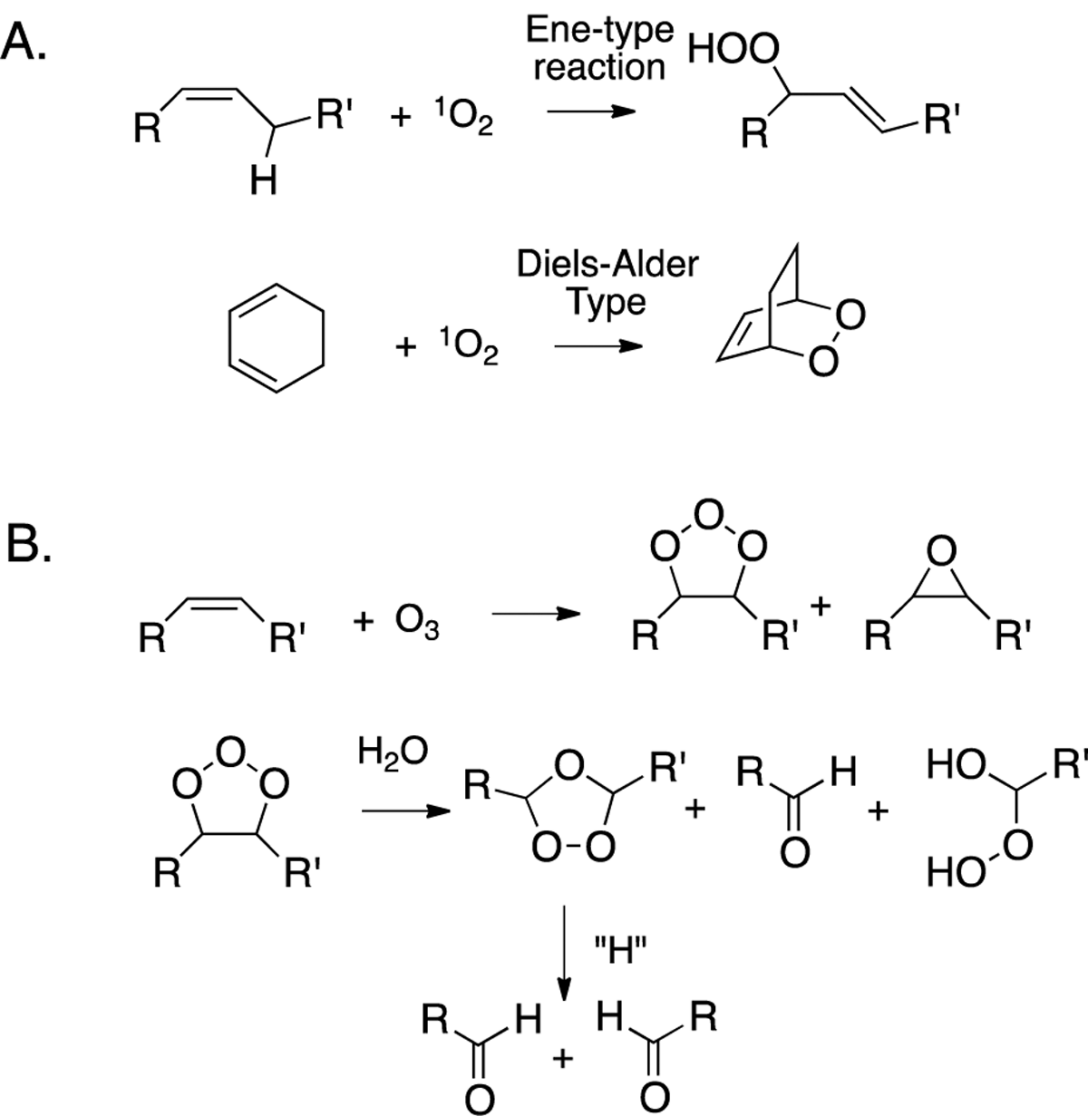
Common reactions of olefins with (**A**) singlet oxygen and (**B**) ozone.

The oxidation of cholesterol by singlet molecular oxygen, ozone, and peroxyl free radicals has been particularly well-studied. While hydroxyl radicals will also undoubtedly react with cholesterol, these species will react with virtually any C-H bond encountered, eliminating these reactions as likely sources of oxysterols in a complex cellular environment. Isolated olefins like the Δ^5,6^ double bond in cholesterol will undergo “ene”-type reactions with singlet oxygen to primarily give chol 5α-OOH, and to a lesser extent, the chol 6-OOHs, and conjugated dienes like those present in 7-DHC give cyclic peroxides by Diels-Alder type transformations (see Figure 2A). Ozone’s primary reaction with isolated double bonds is a [3+2] cycloaddition, producing an unstable adduct (ozonide). Subsequent decomposition of this (primary) ozonide eventually results in carbonyls as the major products, although, under some conditions, α-substituted hydroperoxides are formed, as are epoxides (Figure 2B). The link between primary ozone exposure and deleterious health consequences has been a topic of continued interest. When inhaled, ozone reacts with cholesterol in airway epithelial cells by mechanisms such as those presented in Figure 2B [70–74].

**Figure 3.**
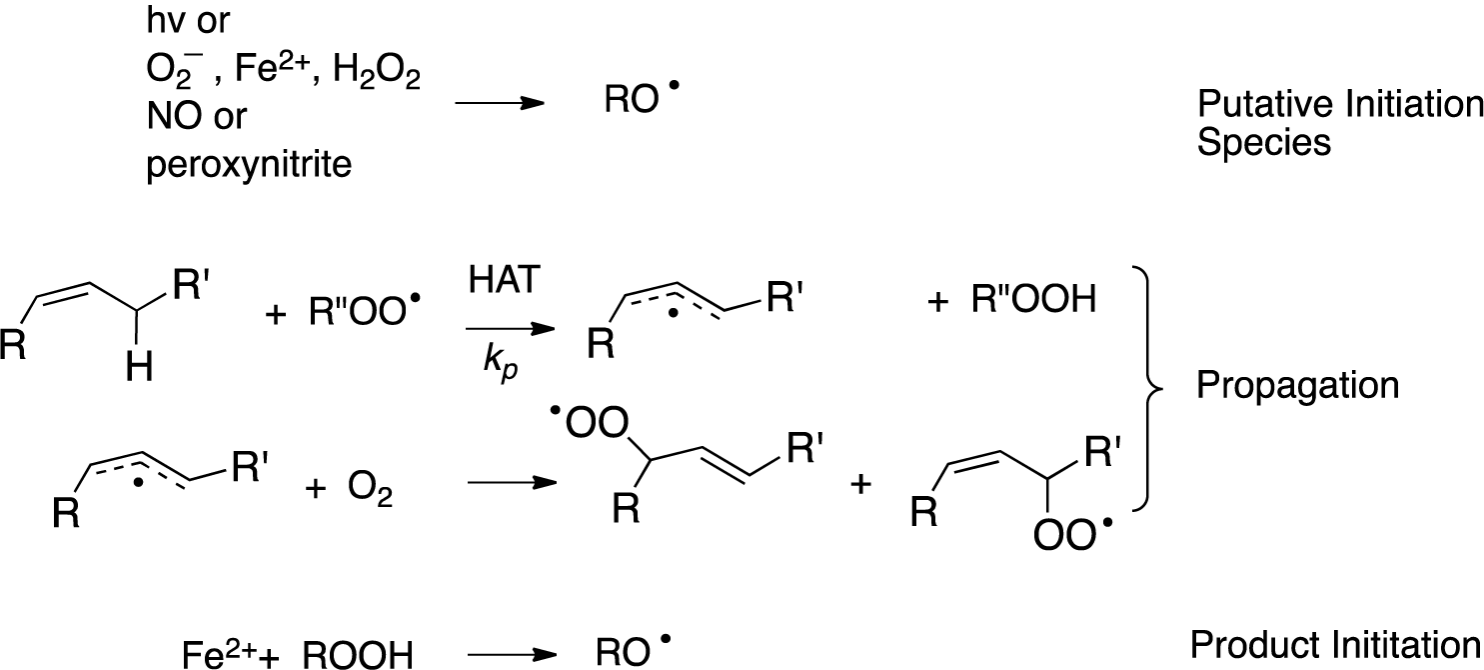
Mechanisms of initiation and propagation for free radical chain oxidation (peroxidation or autoxidation). Propagation steps are illustrated with an isolated alkene reactive substructure.

Free radical peroxidation (autoxidation) is a chain reaction mediated by peroxyl free radicals. The two primary propagation steps in the process are rate-limiting hydrogen atom transfer (HAT) from an organic substrate to a peroxyl radical and the near diffusion controlled addition of oxygen to an intermediate carbon radical (see Figure 3) [75]. The events that initiate the chain sequence have been of interest for as long as the process has been studied with a general understanding that the peroxide products of the reaction can themselves serve as initiators of the process. As a consequence, a single radical-generating event can lead to the cascade formation of peroxide initiators and a dramatic increase in the rate of “auto” oxidation.

The nature of important radical-generating events in biology has been the topic of some debate, with McCord’s suggestion of a metal-mediated Haber–Weiss reaction [76] and the iron-promoted decomposition of lipid hydroperoxides [77–81] having been suggested some four decades ago. The latter has gained prominence with the recent characterization of ferroptosis as the oxidative cell death modality associated with the accumulation of lipid peroxidation products [17–19].

## 3. Cholesterol Autoxidation

**Figure 4.**
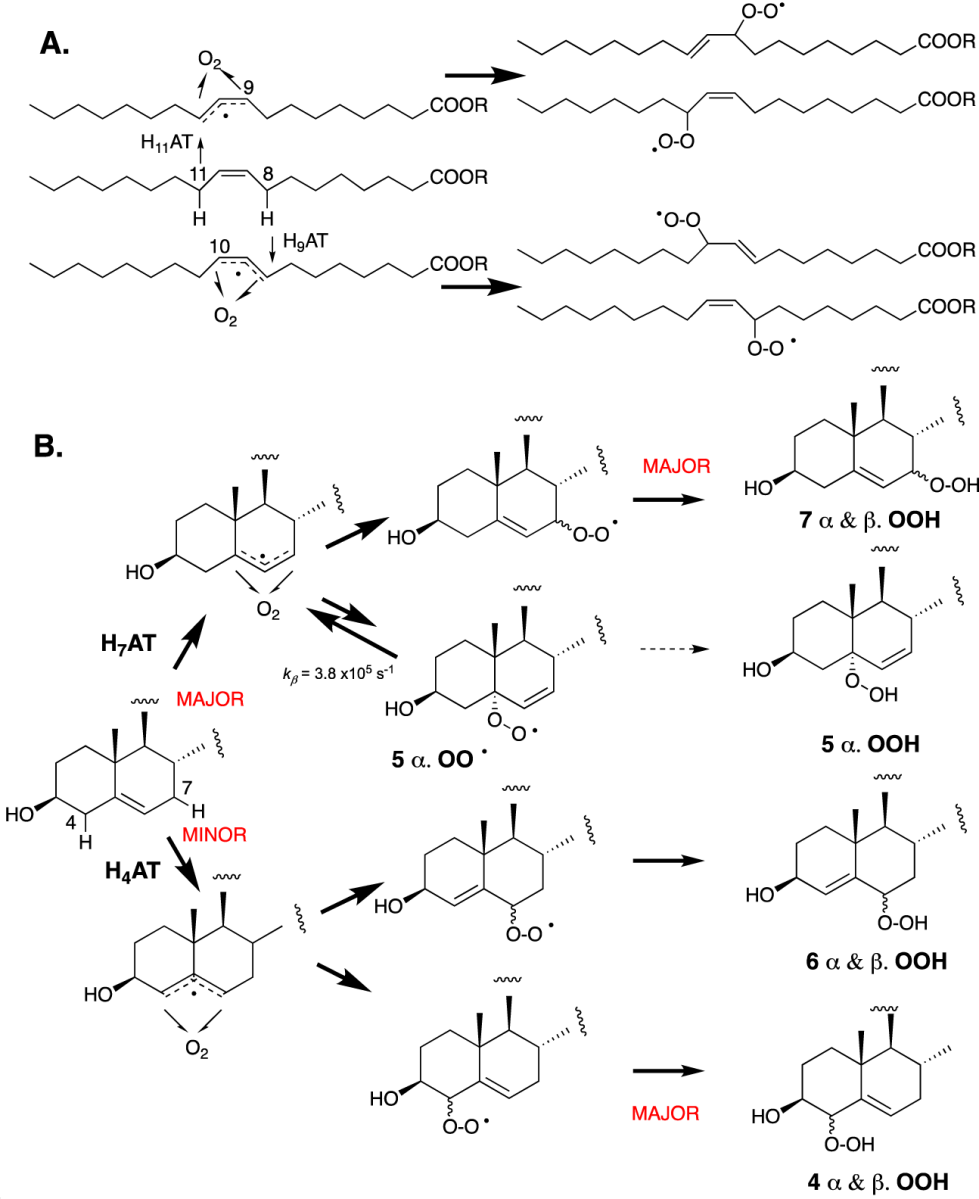
Propagation mechanisms for the autoxidation of (**A**) oleate, where H-atoms at C8 and C11 have similar reactivities toward hydrogen atom transfer (HAT), and (**B**) cholesterol, where H-atoms at C7 are significantly more reactive than C4 hydrogens.

As a monounsaturated lipid, cholesterol may be expected to autoxidize by a mechanism analogous to that of oleic acid—the prototypical monounsaturated fatty acid. Oleic acid autoxidizes via an analogous mechanism to more highly unsaturated fatty acids (vide infra, Figure 4). Specifically, H-atom abstraction from one of the two allylic positions (C8 or C11) leads to two isomeric allylic radicals to which O_2_ can add, and the resultant peroxyl radicals propagate the chain reaction to yield a mixture of regio- and stereoisomeric hydroperoxides [82]. The autoxidation chemistry of cholesterol differs from that of oleic acid because the reactivity of the two allylic positions is not identical. The proximity of the electronegative 3-OH substituent and the integration of the allylic positions in two distinct carbocyclic rings have a substantial impact on both the rates of H-atom abstraction and the stability of the peroxyl radicals that are formed upon the addition of O_2_ to the resultant allylic radicals. In oleic acid, the electronegative carboxylic acid moiety is seven carbon atoms away and both allylic positions are part of acyclic aliphatic carbon chains, such that they are essentially identical in reactivity.

The C-H bond dissociation enthalpies (BDEs) of the two allylic positions at C4 and C7 have been computed to be 89.0 and 83.2 kcal/mol, respectively, whereas the allylic positions in oleate have been computed to have a C-H BDE value of 83.4 kcal/mol [41]. The stronger bond at C4 results from (1) the electron-withdrawing effect of the neighboring C3-OH, (2) the lower substitution of the terminal ends of the allylic radical where the unpaired electron spin density is localized, and (3) the associated planarization of both the A and B rings of the steroid required to maximize radical delocalization. As a result, H-atom abstraction from C7 is favored by a significant margin. The resultant allylic radical can, in principle, be oxygenated at C5 and C7 to yield peroxyl radicals that can propagate the chain reaction, However, since the C5-peroxyl radical is relatively unstable, it generally undergoes rapid β-fragmentation instead of propagation, leaving the C7-peroxyl-derived product, C7-OOH, as the major autoxidation product. C4-OOH and C6-OOH resulting from initial H-atom abstraction from C4 are also formed in measurable amounts because H-atom abstraction from C4 is faster than predicted by its relatively strong bond due to favorable interactions in the H-atom transfer transition state [41].

Radical trapping antioxidants (RTAs) can have a profound effect on the profile of peroxide products formed in the course of inhibited free radical lipid oxidations [83–86]. The autoxidation of cholesterol in solution and in the presence of high concentrations of a very good H-atom donor, such as pentamethylchromanol (PMC), which is a truncated version of α-tocopherol, yields a significant amount of C5α-OOH [39,41]. PMC traps peroxyl radicals in solution with a rate constant in excess of 10^6^ M^−1^ s^−1^, making hydroperoxide formation from C5α-OO^•^ competitive with β-fragmentation—a unimolecular process that occurs with a rate constant of *k*_β_ = 3.8 × 10^5^ s^−1^. No C5β-OOH is observed, presumably due to a much larger *k*_β_ of the precursor peroxyl radical arising from repulsion from the C9β-methyl substituent.

**Figure 5.**
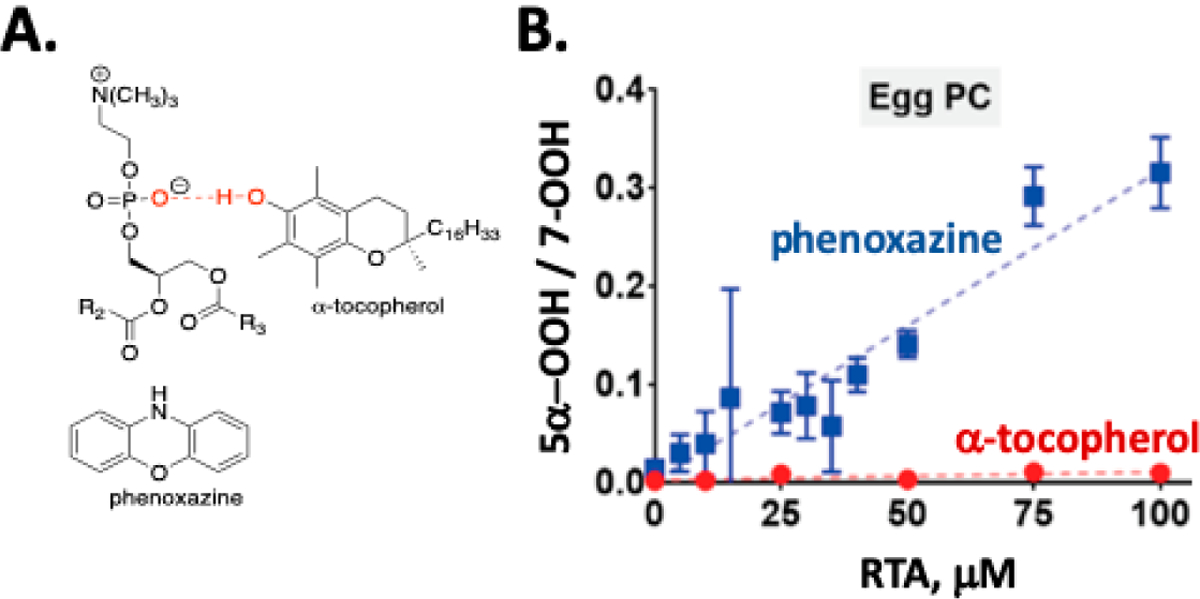
(**A**) Hydrogen bonding in membrane bilayers reduces the effect of phenolic antioxidants like α-tocopherol. (**B**) Phenoxazine scavenges 5α-OOH in bilayers, while α-tocopherol does not.

The autoxidation of cholesterol incorporated into phosphatidylcholine liposomes gives the same set of products that are found in solution, but the dynamics of reactions in membrane-like vesicles have a significant effect on the product profiles observed when compared to the product distributions found in isotropic solutions. One notable feature of cholesterol autoxidation in phosphatidylcholine liposome is that no evidence of C5α-OOH formation was found when α-tocopherol or PMC was incorporated into the vesicles [40]. This can be understood when consideration is given to the fact that α-tocopherol is a much poorer H-atom donor in phospholipid bilayers (*k*_inh_ = 4.7 × 10^3^ M^−1^ s^−1^) compared to organic solutions (*k*_inh_ = 3.6 × 10^6^ M^−1^ s^−1^) due to phenolic hydrogen bonding with the polar headgroup of the phospholipid (see Figure 5A) [87]. Phenolic RTAs have thus been proven to be much less effective at trapping short-lived peroxyl radicals in membrane-like vesicles than in isotropic media, which is a conclusion that may have consequences for the ultimate success of phenolic antioxidant therapies. In contrast to the results obtained with α-tocopherol, the use of aromatic amine RTAs, such as phenoxazine [87], in the oxidation of cholesterol in liposomes leads to the formation of C5α-OOH (see Figure 5B). Phenoxazine has an apparent rate constant for reactions with peroxyl radicals in liposomes (*k*_inh_ = 2.4 × 10^5^ M^−1^ s^−1^) that is some 50 times greater than α-tocopherol, making H-atom transfer from the amine competitive with β-fragmentation of the C5α peroxyl radical [40].

In competition with H-abstraction from the allylic positions in cholesterol, peroxyl radicals add to the C5–C6 double bond. Addition yields a short-lived alkyl radical that can undergo an intramolecular homolytic substitution (*S*_H_^i^) reaction on the peroxide moiety to yield an epoxide or undergo O_2_ addition to produce a different chain-propagating peroxyl radical. Epoxides make up a significant fraction of the product mixture formed in autoxidation, with the a:β epoxide ratio of ~3:1 having been found. Epoxide formation can be eliminated by antioxidants, since the process involves an intermolecular addition of the peroxyl radical to cholesterol. Antioxidants trap peroxyl radicals, completely suppressing the radical addition pathway, while the formation of product hydroperoxides is reduced, but not eliminated. Antioxidant-mediated peroxidation has been suggested to account for the differential effect of antioxidants on epoxide and hydroperoxide products [41]. The tocopheryl radical, for example, can mediate peroxidation by H-atom abstraction, but it cannot facilitate reactions leading to epoxides.

## 4. Cholesterol-Derived Electrophiles

**Figure 6.**
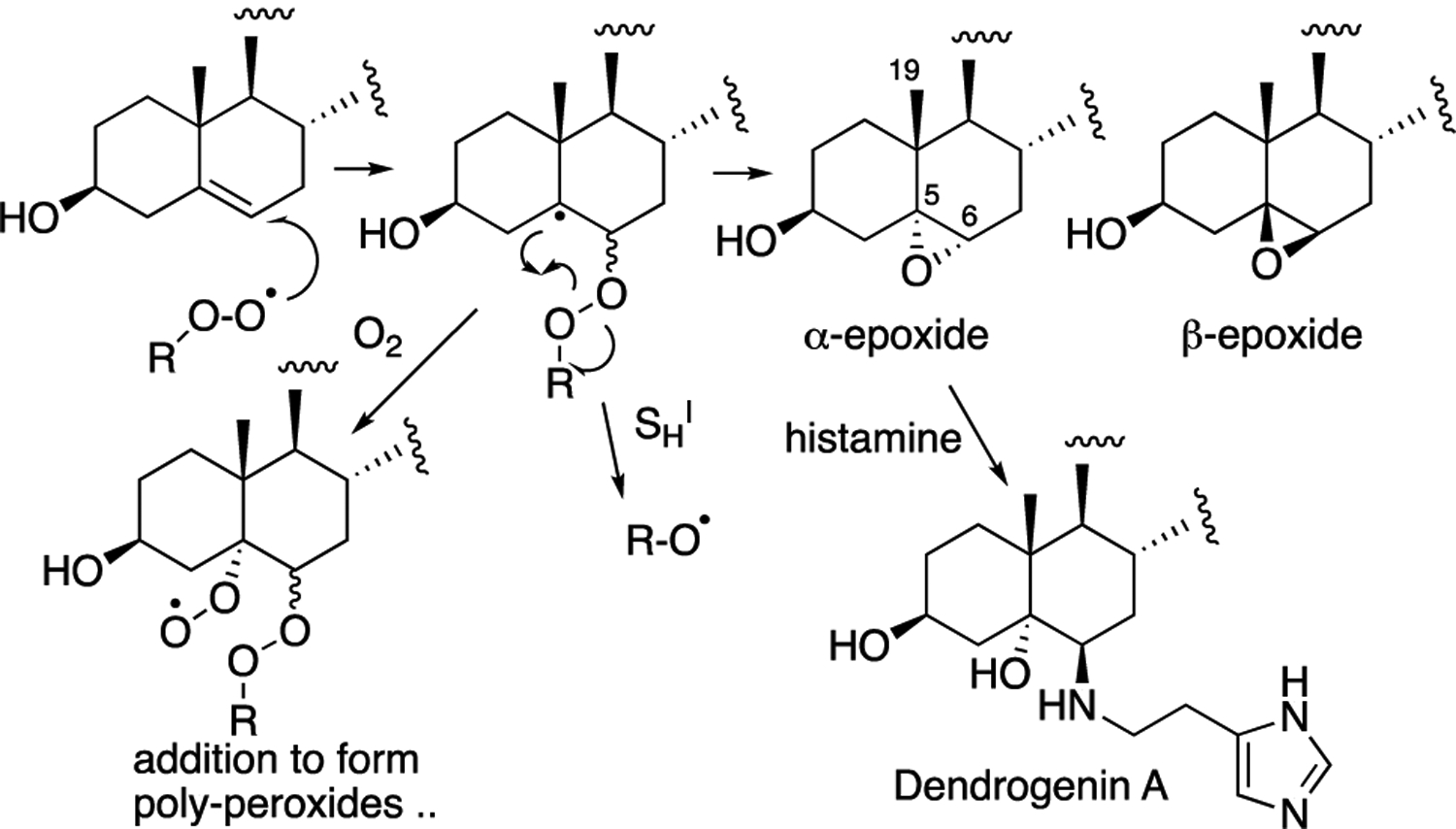
Epoxide formation and ring opening reactions. Intermolecular addition of a peroxyl radical followed by intramolecular homolytic substitution (S_H_^i^) attack of the intermediate carbon radical on the peroxide gives an alkoxyl and the α- and β-epoxides. Oxygen addition can compete with S_H_^i^. Dendrogenin A is a product of histamine and the α-epoxide.

Although epoxides are nominally electrophilic, the cholesterol 5,6-epoxide is too highly substituted for efficient nucleophilic substitution. C5 is fully substituted and thus unreactive to bimolecular nucleophilic substitution (*S*_N_2) and, although C6 is a tertiary center, the adjacent fully substituted C5 and the C19β-methyl substituent hinder the addition of nucleophiles to the α-epoxide, while the adjacent axial C-H bond at C9 hinders the addition to the β-epoxide [88]. The epoxides are, however, substrates for hydrolase-catalyzed ring-opening hydration [89–93], as well as non-enzymatic reactions with thiol and amine nucleophiles [89,94–101]. Dendrogenin A, the product of the enzymically-promoted ring-opening reaction of the α-epoxide with histamine shown in Figure 6, was recently discovered in mammalian tissues [94–101]. This sterol alkaloid has a specific potency with regards to inducing cell differentiation at low doses, suggesting its possible existence as a cholesterol metabolite.

Dehydration or further oxidation of the cholesterol α- and β-stereoisomers of C4-OOH, C6-OOH, and C7-OOH yields the corresponding ketones. Although these are α,β-unsaturated carbonyls—the quintessential motif of Michael acceptors—studies with the most abundant of these, 7-ketocholesterol, have indicated that it is not particularly electrophilic [102]. It remains unclear whether sufficient levels of 4-ketocholesterol or 6-ketocholesterol are formed in vivo to be relevant. However, should they be, they are expected to be more reactive than 7-ketocholesterol, because the β-carbons are monosubstituted and less sterically hindered than the disubstituted and sterically-hindered β-carbon in 7-ketocholesterol.

The fragmentation of cholesterol hydroperoxides leads to highly electrophilic aldehydic species. Both C5α-OOH and C6β-OOH readily undergo acid-catalyzed (Hock) fragmentation [103–105] to give the B-ring cleavage product secosterol A, and its aldolized product, secosterol B (see Figure 6) [39–41,106]. These derivatives are precisely the same compounds as those formed from the reaction of cholesterol with ozone [70,71,74,107,108]; their character as reactive electrophiles will be subsequently discussed in this review. The C5α-OOH and C6β-OOH compounds are minor products in the free radical oxidation of cholesterol compared to products formed with hydroperoxide functionality at C7, so the propensity for Hock fragmentation and the products derived from the C7 hydroperoxides are of interest.

**Figure 7.**
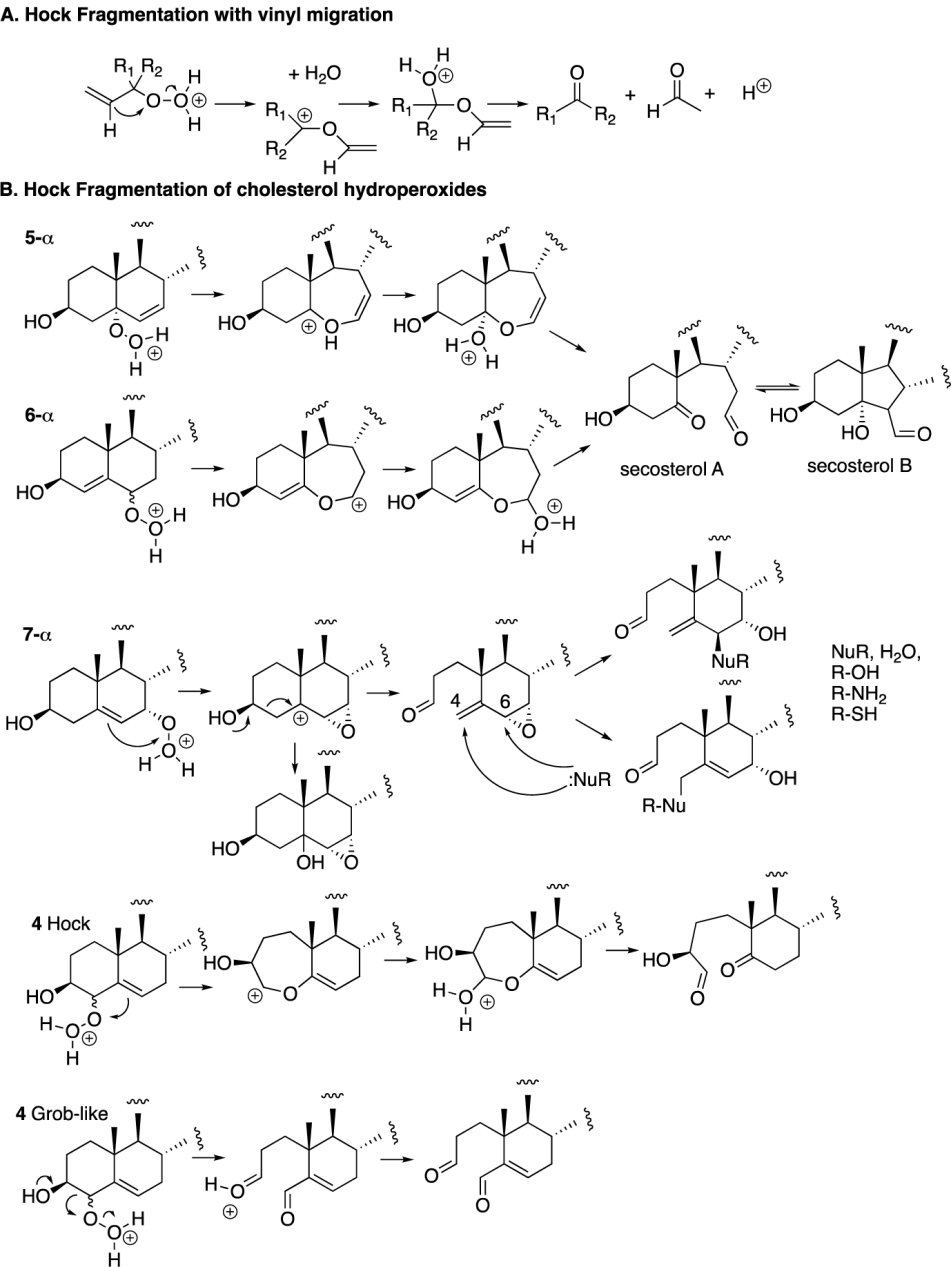
Hock fragmentation mechanisms. (**A**) General mechanism for the acid-promoted fragmentation of allylic hydroperoxides. (**B**) Acid-catalyzed fragmentation of cholesterol hydroperoxides.

Recent studies showed that C7α-OOH undergoes Hock fragmentation readily, while C7β-OOH is unreactive [40]. This rearrangement does not follow the typical Hock mechanism shown in Figure 7A; instead, an intermediate epoxy carbocation is formed, followed by water entrapment or fragmentation, to give an allylic epoxide that should be highly electrophilic. Indeed, under the conditions of Hock treatment and workup in the presence of ethanol, epoxide hydrolysis and/or ethanolysis products are observed, as shown in the figure. Presumably, amine and thiol nucleophiles would add to either the C4 or C6 centers, opening the possibility of protein adduction from this reactive species. The C4-OOH compound is a minor product of free radical oxidation and the identities of the products formed from this precursor have yet to be reported. Potential pathways for the reaction of C4-OOH under Hock-like conditions are suggested in Figure 6. The Grob-like pathway outlined in the figure would provide an electrophilic di-carbonyl compound similar to secosterol A that would adduct to proteins in either aldol form.

It is noteworthy that glutathione peroxidase-4 (GPX4), which is primarily responsible for the detoxification of lipid hydroperoxides [109], reacts with the different regio- and stereoisomers of cholesterol hydroperoxides at different rates (i.e., 5α-OOH < 6αR-OOH ≈ 7α/β7-OOH < 6β-OOH) [110]. As such, although 5α-OOH may form at a slower rate than the other regio/stereoisomers, it may accumulate due to its slower reduction by GPX4, and fragment to afford secosterol A and B. To the best of our knowledge, the relative reactivity of C4-OOHs as substrates for GPX4 has not yet been determined.

## 5. Autoxidation of 7- and 8-Dehydrocholesterols

**Figure 8.**
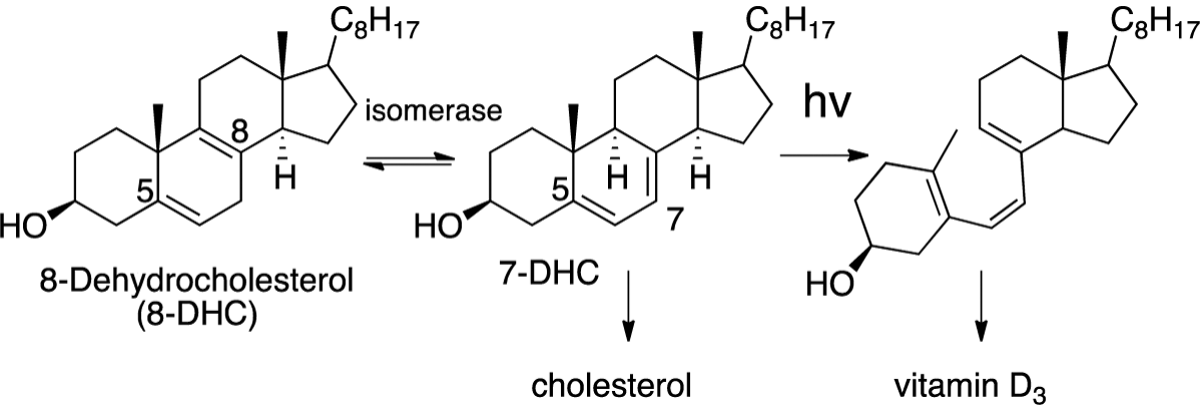
7-dehydrocholesterol (7-DHC) is a biosynthetic branchpoint between cholesterol and vitamin D_3_; 7- and 8-DHC are equilibrated by an isomerase enzyme.

7-DHC, the immediate biosynthetic precursor to cholesterol, is usually found in human tissues and fluids at very low levels compared to cholesterol. The isomer 8-DHC, which has a homo-conjugated 5,8-diene [111], is found at comparably low concentrations in tissues and fluids, with an isomerase (EBP) able to interconvert them. 7-DHC occupies a branchpoint in isoprenoid biosynthesis between cholesterol and vitamin D_3_ [112]—the B-ring diene undergoing a photochemically-promoted ring opening on the pathway to the vitamin (see Figure 8) [113,114].

The autoxidation of 7- and 8-DHC has drawn increased attention in recent years [61,62], but the susceptibility to the oxidation of ergosterol, which is a 5,7-diene analog of 7-DHC found in fungi and protozoa, was noted over a century ago [115–117]. The 1933 publication of Meyer on ergosterol is particularly noteworthy [117]. Three mechanisms for oxidation were suggested, with two involving photolysis in the presence or absence of dyes that likely involve the intermediacy of singlet molecular oxygen. A third mechanism that has all of the characteristics of a free radical chain reaction consuming over 2 moles of oxygen per ergosterol, is promoted by heme in the dark and is diminished if ergosterol is carefully purified, thus removing peroxides that could initiate autoxidation.

**Figure 9.**
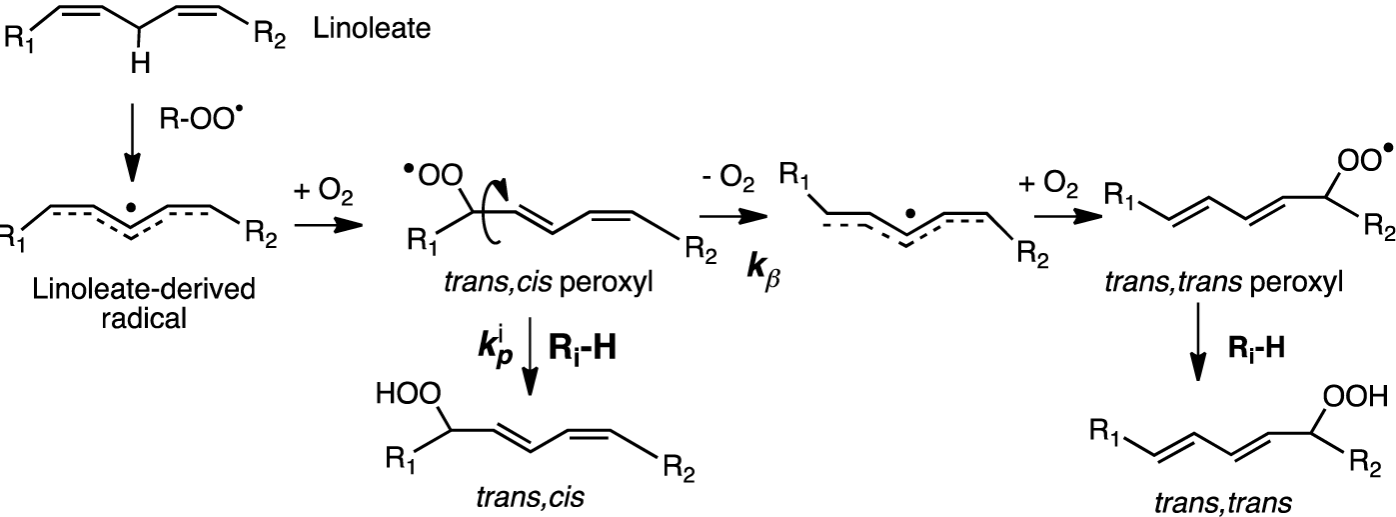
Linoleate oxidation mechanism and basis of a free radical clock for determining the propagation rate constant ***k***_***p***_ for the autoxidation of any **R**_**i**_**-H**.

Nearly 50 years after Meyer, the enhanced susceptibility of 7-DHC to oxidation was noted in studies of its free radical co-oxidation in liposomes made up of unsaturated linoleate phospholipids [118]. Linoleate gives *trans-cis* and *trans-trans* conjugated dienes under conditions of free radical chain oxidation and the ratio of these products was found to reflect the H-donor character of the medium undergoing oxidation (see Figure 9) [119]. The oxidation of liposomes of phospholipids bearing a linoleate and a palmitate on the glyceryl headgroup, for example, gave linoleate conjugated diene products at a *trans-cis*/*trans-trans* ratio of 0.69, but if the liposomes were made up as a mixture of 0.7 moles of the linoleate phospholipid to 0.3 moles of 7-DHC, the *trans-cis*/*trans-trans* ratio determined was nearly 3.0. In the presence of good H-atom donors, more *trans-cis* products were formed, and in the absence of H-atom donors, more *trans-trans* products were found.

The linoleate mechanism describes a unimolecular process—the loss of oxygen from the *trans-cis* peroxyl (***k*_*β*_**) in competition with bimolecular H-atom transfer from **R**_**i**_**-H** to a peroxyl radical that occurs with rate constant ***k***_***p***_. This kinetic competition is the basis for a radical clock approach to determine propagation rate constants for the autoxidation of a number of small molecules, fatty esters, and sterols [61,83,120]. The rate constant originally determined in benzene at 37 °C for cholesterol by the clock method was 11 M^−1^ s^−1^, and a more recent value of 8.4 M^−1^ s^−1^ confirms that cholesterol is roughly 10-times more reactive than oleic acid [40]. These rate constants represent the sum of the values for H-atom transfer from C-7 and C-4 of the molecule, although, since C7-derived products dominate HAT, from C7, it is presumably much faster than from C4, as predicted by computations. For comparison, the ***k***_**p**_ of 7-DHC was found to be ca. 2260 M^−1^ s^−1^, some 200 times the cholesterol value. Indeed, 7-DHC has the largest rate constant for free radical propagation found to date for a lipid molecule. The values of ***k***_**p**_ determined for other lipids of interest were 197 M^−1^ s^−1^ for arachidonate and 960 M^−1^ s^−1^ for 8-DHC [121]. The kinetics of the autoxidation of linoleate phospholipid liposomes has also been examined and the relative reactivity of lipids studied in isotropic media is mirrored in liposomal bilayers, with 7-DHC having the greatest effect on the linoleate hydroperoxide *trans-cis*/*trans-trans* product ratio of any oxidizable co-substrate [61].

**Figure 10.**
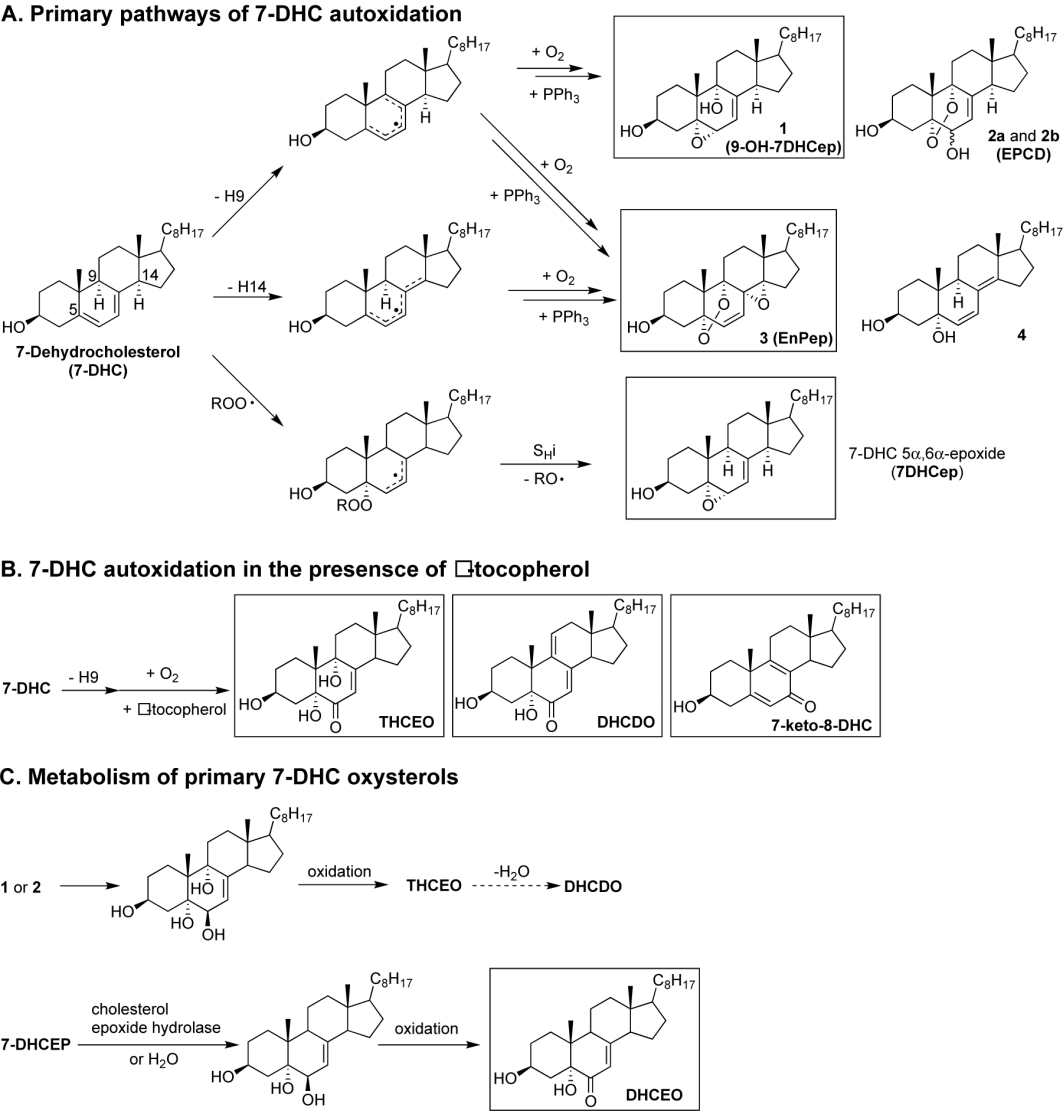
Mechanisms of the formation of 7-DHC-derived oxysterols in solution (**A**,**B**) and in biological systems (**C**). (**A**) Primary pathways of 7-DHC autoxidation in organic solution at 37 °C; (**B**) 7-DHC autoxidation in the presence of α-tocopherol (only loss of the H-9 pathway is shown); (**C**) metabolism of primary 7-DHC oxysterols in cells. Compounds highlighted in the rectangle are potential electrophiles.

Studies on the products formed from the autoxidation of 7-DHC suggest that it is reactive as both an H-atom donor and a peroxyl radical addition acceptor [29,61,67]. Based on product and mechanistic studies, the high reactivity of 7-DHC was rationalized by the planarity of the conjugated system, the perfectly aligned allylic C-H bonds at C9 and C14 for hydrogen abstraction, the highly substituted pentadienyl radical after H-atom removal, and the formation of a stabilized allylic radical after peroxyl radical addition. Therefore, both H-atom transfer (loss of the H-atom at C9 or C14) and peroxyl radical addition (to the conjugated diene) contributes to the free radical oxidation of 7-DHC (Figure 10). The loss of H-9 from 7-DHC (or H-7 from 8-DHC) leads to a pentadienyl radical in ring-B, which then undergoes oxygen addition [122] and a series of intramolecular radical rearrangements to give a number of oxysterol products, including compounds **1** (5α,6α-epoxycholest-7-en-3β,9α- diol or 9-OH-7DHCep), **2a** (5,9-endoperoxy-cholest-7-en-3β,6α(β)-diol or EPCD-a), **2b** (EPCD-b), and **3** (5α,9α-Epidioxy-8α,14α-epoxycholesta-6-en-3β-ol or EnPep). The loss of H-14 leads to a pentadienyl radical across rings B and C and eventually to compounds **3** and **4**, as major products. On the other hand, peroxyl radical addition to 7-DHC results in the formation of 7-DHC 5α,6α-epoxide (7DHCep).

As was the case for cholesterol, when the oxidation of 7-DHC was carried out in the presence of tocopherol, the peroxyl radical addition pathway was completely suppressed while other oxidation products were still observed, most likely by tocopheryl-mediated H-atom transfer. The abstraction of H-9 leads to the formation of a number of oxysterols containing the enone moiety, including 3β,5α,9α-trihydroxycholest-7-en-6-one (THCEO), 3β,5α-dihydroxycholesta-7,9(11)-dien-6-one (DHCDO), and 7-keto-8-dehydrocholesterol (7-keto-8-DHC), and the H-14 pathway gives peroxyl radicals that lead to simple dienol products (not shown) [121]. It should be noted that compounds **1**–**4** have not been observed in cell and animal models of SLOS, but metabolites of **1**, **2a**, and **2b**, including THCEO and DHCDO, have been observed in vivo along with 7-keto-8-DHC. Furthermore, a metabolite of 7DHCep, 3β,5α-dihydroxycholest-7-en-6-one(DHCEO),has been observed at high levels in cell and animal models of SLOS, particularly in fibroblasts and mice brains [68,123]. The observation of DHCEO is interesting because it suggests that the level and distribution of α-tocopherol in vivo is not sufficient to completely suppress the peroxyl radical addition pathway.

## 6. 7- and 8-Dehydrocholesterol-Derived Electrophiles

The oxysterols **1** and 7DHCep derived from 7- and 8-DHC oxidation are excellent electrophiles based on the presence of their substructure allylic epoxide moiety, while DHCEO, THCEO, and DHCDO may also be electrophiles due to their α,β-unsaturated enone. Indeed, evidence will be presented in a subsequent section of this review that 7DHCep or 7-DHC readily adduct proteins. Compound **1** appears to be as reactive as 7DHCep toward nucleophilic adduction, while the enone-containing oxysterols, DHCEO and THCEO, are hindered toward Michael addition due to the γ-alkyl substituents and the axial C-H or C-OH bonds at C9 and C14, while such addition for DHCDO is less hindered and a likely Michael acceptor. Although all of these enone moieties can potentially form imine adducts with protein lysine residues, this reactivity typically requires acid catalysis and is reversible.

7-DHC-derived oxysterols exert varied cytotoxicity in vitro that is dependent on their specific structures. Specifically, oxysterols EPCD-a and -b are toxic in Neuro2a cells and retina-derived cell lines, with EnPep and DHCEO showing a lower toxicity in these cells [124,125]. Among all of the cells tested, primary neurons appear to be the most susceptible to oxysterol exposure, with an IC50 of approximately 0.75 μM for DHCEO—the most abundant autoxidation-derived oxysterol observed in the brain of SLOS rodent models. Interestingly, at a physiological concentration (5 μM) [63], DHCEO accelerates the formation of neuronal processes from primary neurons, such as dendrites [68]. On the other hand, the electrophilic 9-OH-7DHCep (compound **1**) did not display any toxicity to Neuro2a cells, indicating that electrophilicity may not be the most important determinant of cytotoxicity [124]. Cytotoxicity of the precursor of DHCEO, 7DHCep, has not been examined, but a large number of protein adducts with this oxysterol have been reported in *Dhcr7*-knock down Neuro2a cells [69].

It is interesting to note that the concentrations of DHCEO in the brain of SLOS rodent models are much higher than those in the matching liver and retina. For example, in AY9944-treated rats, even after normalization by the levels of 7-DHC (DHCEO/7-DHC), the amount of DHCEO observed in the brain is 2.7-fold of that in the liver and 11-fold of that in the retina. We speculate that several factors could account for such tissue-specific variation. The metabolism of 7DHCep may be tissue-specific, as the ring-opening of the epoxide is likely an enzymatic process based on the precedent of the soluble cholesterol 5,6-epoxide hydrolase (ChEH) [126] More recently, ChEH was identified as a hetero-oligomer of the cholesterol biosynthesis enzymes DHCR7 and 3β-hydroxysterol-Δ^8^-Δ^7^- isomerase (EBP), and the same hetero-oligomer also serves as the antiestrogen binding site (AEBS) [89]. No study has been carried out to determine if 7-DHCep can be a substrate of ChEH, but an earlier report concluded that 7-DHC 5β,6β-epoxide can serve as a mechanism-based inhibitor of rat microsomal ChEH via covalent modification of the active site by the reaction intermediate [91]. 7DHCep (epoxide on the α-face of the sterol ring) was not tested in that study, but the stereochemistry of the C5-hydroxyl group of the cationic intermediate resulting from α- or β-epoxide could potentially lead to different fates of this intermediate, i.e., covalent modification of the enzyme active site vs. nucleophilic attack by water to give the diol on C5 and C6.

It should also be noted that the amount of DHCEO is likely dependent on the extent of reactions between 7DHCep and nucleophiles in a particular tissue. Therefore, nucleophiles could be protein residues or glutathione (GSH), which is an abundant nucleophile in some tissues. The more adduction of 7DHCep that occurs in any organ, the less DHCEO that is found in that tissue. GSH conjugation with electrophiles is normally catalyzed by glutathione S-transferases (GST) [127]. Indeed, a rat microsomal GST (isoform B, which is equivalent to GST A1 in current nomenclature) has been found to catalyze the conjugation between cholesterol 5α,6α-epoxide and GSH [93,128].

It is known that the human liver contains much higher (2.6-fold) levels of various isoforms of GSTs (with A1 as the predominant isoform), than those in the human brain (with P1 being the major isoform, followed by M3 and M2) [127], which indicates that 7DHCep could be more readily detoxified in the liver than in the brain. The levels of GSTs in the human retina have not been reported, but the GST isoform M1 is expressed at the highest level in photoreceptor cells in the rat retina, where most of the degeneration occurs in the AY9944-rat model, followed by isoforms A4 and P1. Therefore, the variation in the level of DHCEO could arise from the different expression patterns and levels of GST isoforms in each tissue.

Although the level of DHCEO in the retina is low and other 7-DHC autoxidation-derived oxysterols were not observed, retinal degeneration and increased lipid peroxidation are hallmarks of the AY9944-rat model of SLOS, suggesting that the protein adduction of electrophilic oxysterols could be a significant factor contributing to the retinal pathophysiology. Indeed, in a recent pre-clinical therapeutic study using the AY9944-rat model, a combination of cholesterol and antioxidant (vitamin E, vitamin C, and selenite) completely prevented retinal degeneration in this model, while cholesterol supplementation alone only partially prevented this phenotype [129]. An analysis of protein-oxysterol adducts with and without antioxidant treatment has not been accomplished due to current limitations in antibody availability and in vivo pull-down methodology, but such a study would presumably reveal whether protein adduction is indeed underlying the retinal degeneration pathobiology. On the other hand, protein adducts with lipid electrophiles, particularly 4-HNE, have been found to be significantly (9-fold) higher in the retinas of AY9944-treated rats than in matching controls [130], supporting the general elevation of lipid peroxidation and protein-lipid electrophile adducts in this SLOS model.

## 7. Protein Adduction of Lipid-Derived Electrophiles

### 7.1. PUFA-Derived Protein Adducts

**Figure 11.**
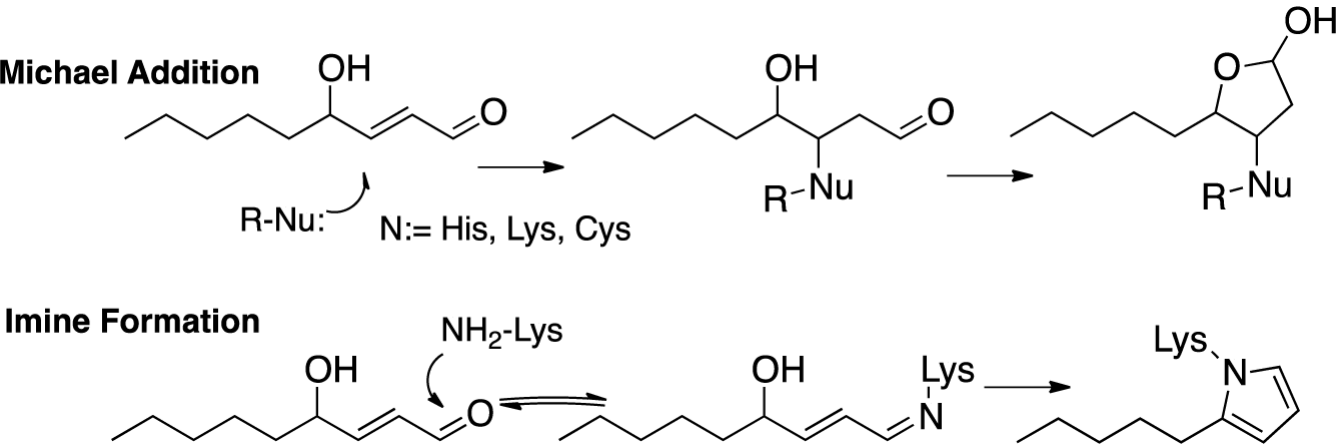
Mechanisms of 4-hydroxy-2-nonenal (4-HNE) protein adduction. Michael-lactol and imine-pyrrole formation.

The formation of protein adducts with fatty acid-derived electrophiles has been the subject of extensive investigations, with 4-HNE (Figure 11) [48,131]—the electrophile generated from the peroxidation of ω-6 fatty acids [132–135]—being a principal focus of interest [49,132–138]. Michael addition of protein cysteines, lysines, or histidines is the most common means of protein covalent attachment to 4-HNE. Lysine also undergoes reversible imine formation that may lead to cyclodehydration with the irreversible formation of a pyrrole protein adduct. Strategies have been developed to isolate and identify protein adducts from PUFA-derived electrophiles [55–57,139–145] and excellent reviews of these topics have been published [57,146,147].

### 7.2. Cholesterol-Derived Protein Adducts

In contrast to the extensive effort to characterize protein modification by 4-HNE and other PUFA-derived species, protein adduction by sterol-derived electrophiles has received much less attention. The reports of Wentworth, Kelly, and collaborators [11,60,148–151] suggested that secosterol electrophiles are present in human atherosclerotic and neurodegenerative tissue, stimulating interest in the field, and subsequent studies showed that protein misfolding is a consequence of protein adduction.

The structural elucidation of protein adducts formed from cholesterol-derived electrophiles has been a topic of extensive research in recent years. While the secosterols have been of particular interest, it should be noted that other cholesterol-derived electrophiles have the same mass as the secosterols (see Figure 7), making the unambiguous structural assignment of adducts found in vivo difficult. For secosterols A and B, the assignment of the structure is further complicated by the fact that these two electrophiles are present in an aldol-retroaldol equilibrium.

**Figure 12.**
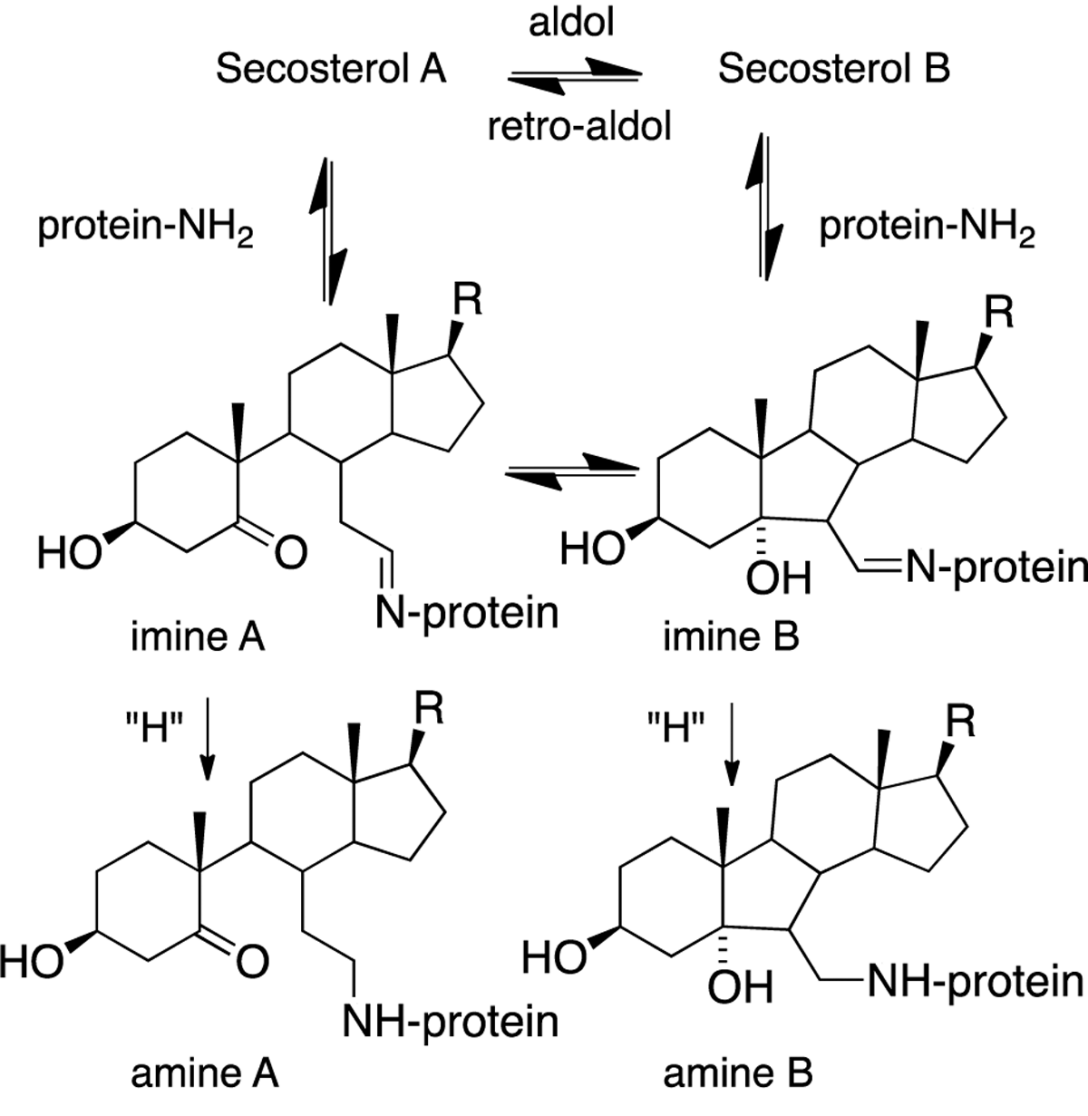
Mechanisms of secosterol-protein reactions resulting in imine reduction to stabilize adducts.

Both secosterol A and B have a free aldehyde that can react with protein lysines (see Figure 12), causing some ambiguity about the nature of the adduct or adduct mixture formed. Reversible imine formation is the presumed initial step of adduction and in most studies of secosterol-protein reactions, imine reduction with borohydride or cyanoborohydride has been used to stabilize the adduct. This reduction strategy is required to “fix” the secosterol-protein covalent bond, since imine bond formation is reversible. The basic conditions of reduction likely minimize this problem; nevertheless, it should not be overlooked. It is also worth emphasizing that the mixture formed after imine reduction is only a close approximation of the authentic adducts formed in a biological setting, with the difference being a labile imine bond for adducts in cells or tissues and a stable sterol protein amine bond after reduction and isolation.

As an example of an important early study of oxysterol conjugates of Alzheimer’s amyloid Aβ-peptides, Usui et. al. used solid phase synthesis to prepare secosterol adducts of specific Aβ peptide lysines, as well as the conjugate of the peptide terminal Asp amine [10]. Reduction of the imine A/B mixture with cyanoborohydride was used to fix the secosterol conjugates at Lys-16 and Lys-28, as well as the N-terminal Asp-1 amine of the Aβ peptide. It is of interest that the secosterol conjugates at Lys-16 and Lys-28 significantly increased the kinetics of Aβ peptide aggregation, while the terminal amine Asp-1 adduct had no measurable effect on the process. The aggregates formed from the Lys-16 secosterol A/B adducts were also found to be highly toxic to cultured cortical neurons.

**Figure 13.**
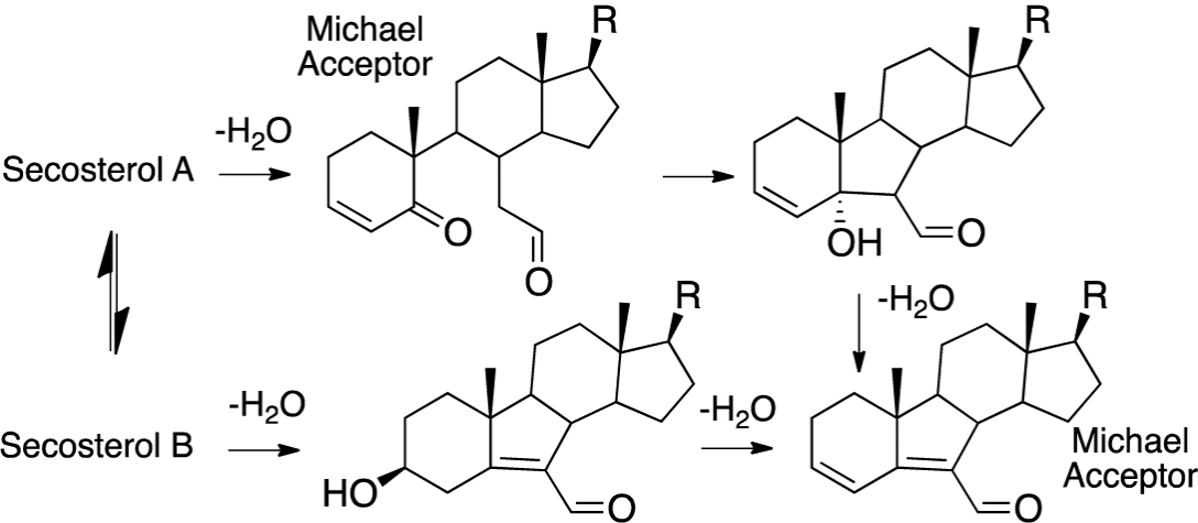
Dehydration of secosterols gives a complex mixture of electrophiles, including two Michael acceptors.

In a detailed study of adduction, Windsor et. al. reported on the reactions of a mixture of secosterol A/B with amino acids, peptides, and isolated proteins [102]. The reaction of secosterol A with lysine under mild conditions (pH 7.4 buffer) gave multiple products, including *m*/*z* = lysine + secosterol A/B, lysine + secosterol A/B-H_2_O, and lysine + secosterol A/B-2H_2_O. Dehydration apparently competes with the aldol cyclization of secosterol A, affording multiple electrophilic species that can react with lysine from the single secosterol A precursor, as shown in Figure 13. Two of the dehydration products have structures that make them likely Michael acceptors and thus capable of adduction with cysteines and histidines and, indeed, histidine adducts are formed when either secosterol A or B is reacted with the model peptide Ac-Ala-Val-Ala-Gly-**His**-Ala-Gly-Ala-Arg.

The exposure of cytochrome c to secosterol A gave evidence of significant adduction, as measured by MALDI-TOF MS analysis of the product mixture, which showed the addition of up to five secosterols to the protein [152]. Following borohydride reduction to fix lysine-derived imines, tryptic digestion and a proteomics assay of the major peptides indicated the presence of both lysine imine and histidine Michael adducts.

**Figure 14.**
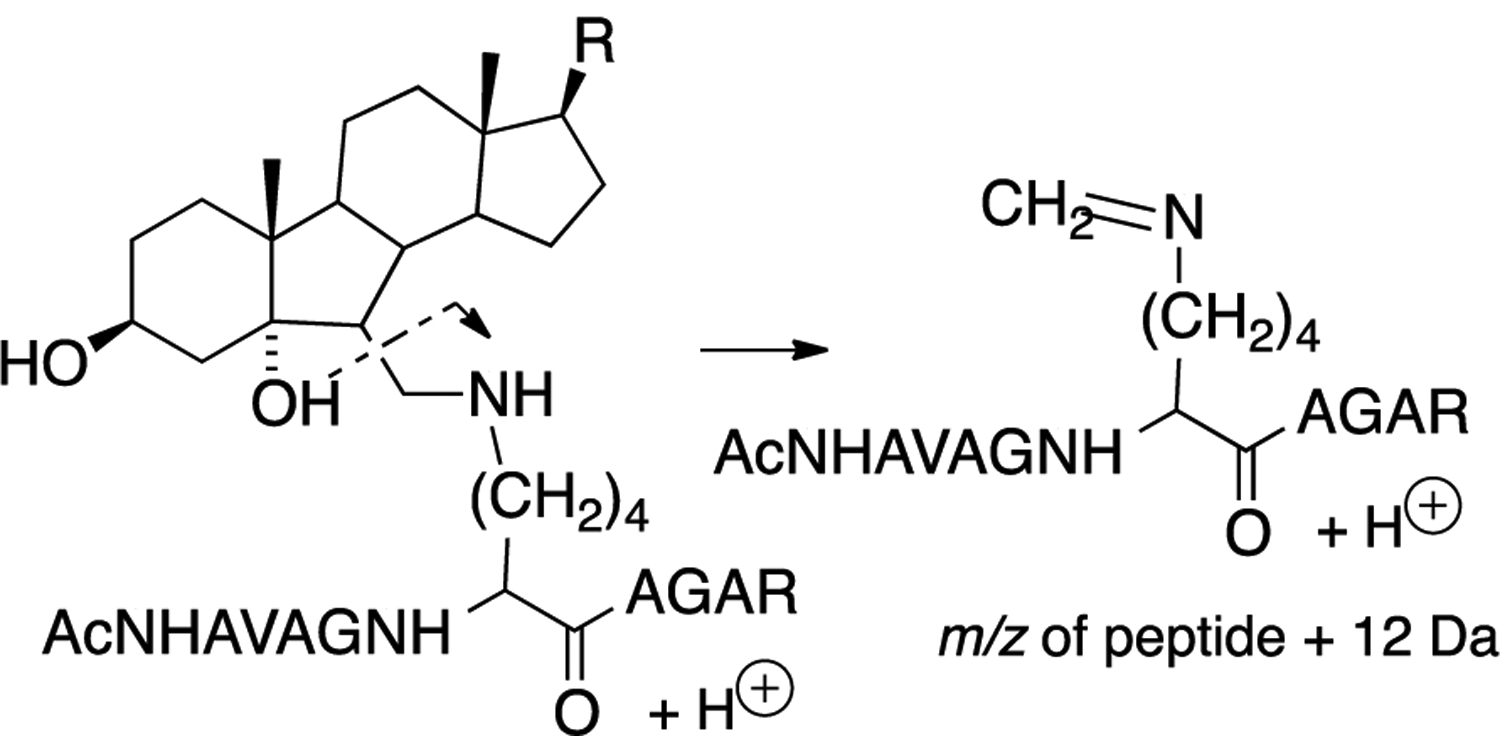
Neutral loss of a sterol fragment gives the tryptic peptide with +12 Da at the modified lysine.

Tryptic peptides of lysine-secosterol adducts undergo characteristic mass fragmentations that have proved useful in determining the specific site of secosterol adduction. This was demonstrated in reactions of secosterol A or B with the model peptide Ac-Ala-Val-Ala-Gly-**Lys**-Ala-Gly-Ala-Arg and is shown in Figure 14, where the neutral loss of a sterol fragment gives the peptide + 12 Da at the site of lysine modification. Subsequent fragmentation of the +12 Da ion gives *b* and *y* ions that indicate the site of the modified lysine on the tryptic peptide, making identification of the protein adduct straightforward.

Histidine adducts having *m*/*z* = peptide + (secosterol A-2H_2_O) at His-33 were found when cytochrome c was reacted with secosterol A in pH 7.4 buffer. This His-33 residue has also been found to be a major site for adduction with the electrophile 4-HNE. Genaro-Mattos et al. reported that when cytochrome c-secosterol A exposure was carried out with micellar SDS present, the major adduct formed was at Lys-22, rather than His-33, which is a result that emphasizes the importance of the protein tertiary structure and nucleophile access [152].

## 8. Alkynyl-Sterol Probes

**Figure 15.**
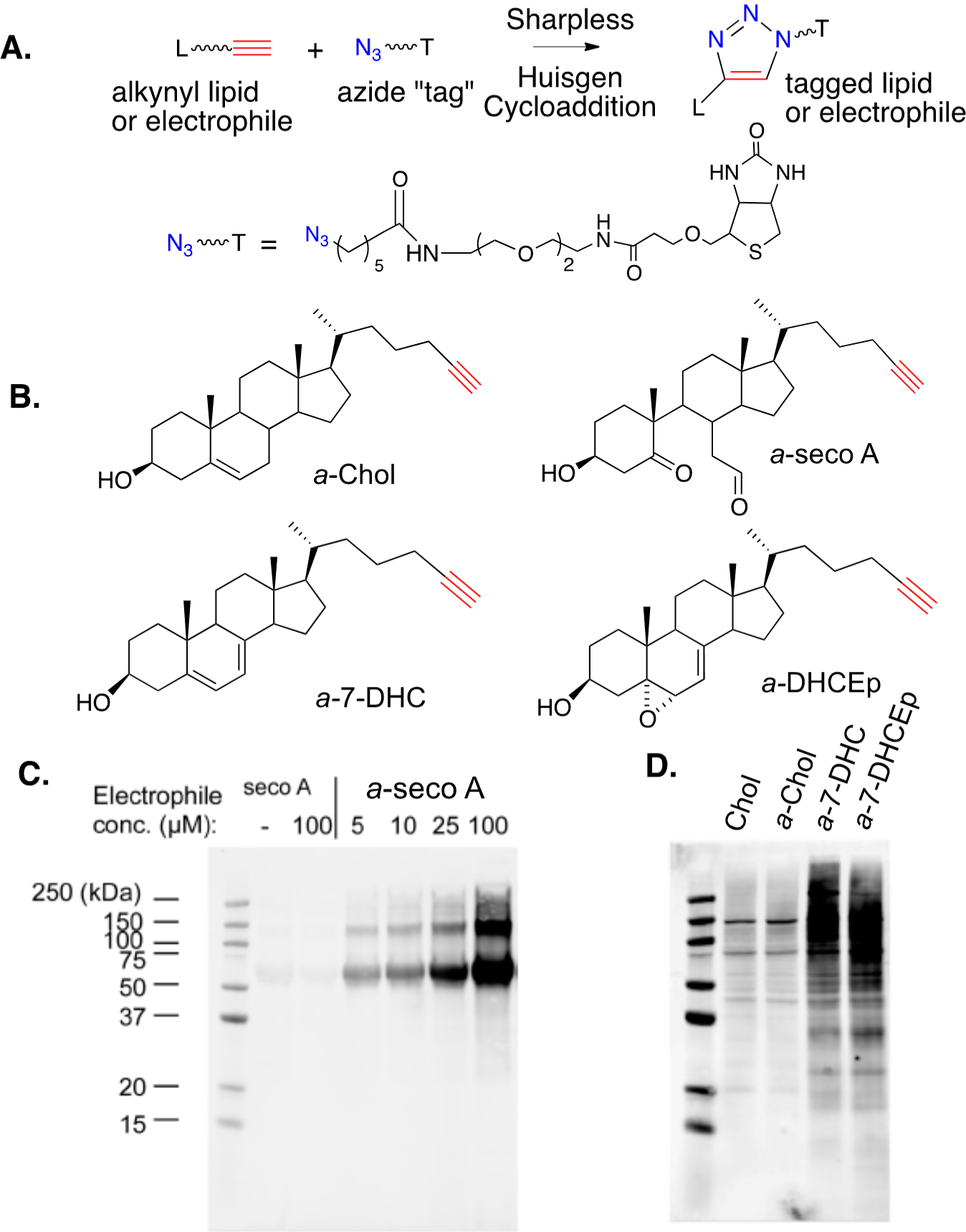
(**A**) Click cycloaddition of an alkynyl lipid and an azide “Tag”. (**B**) Illustrative alkynyl lipids or lipid electrophiles. (**C**) Products of adduction of alkynyl-secosterol A (*a*-seco A) with human serum albumin (HSA) in pH 7.4 buffer, followed by “click” cyclo-addition with an azido-biotin. Gel visualized with a streptavidin fluorophore. (**D**) Protein-lipid adducts of Neuro2a cells treated with 20 μM of cholesterol, *a*-Chol, *a*-7-DHC, and *a*-DHCEp for 24 h.

The development of bio-orthogonal reagents and their use in probing mechanistic pathways and metabolism have seen widespread application in recent years. Sharpless–Huisgen or “click” cycloaddition [153,154] (see Figure 15A), has been applied in a variety of settings to monitor cellular processes and the application of this strategy in studies of electrophile-protein adduction has been particularly useful. Alkynyl versions of PUFAs and their derived electrophiles, including 4-HNE, have been prepared and their use as surrogates for endogenous species has been employed for over a decade [55–57,139–145]. The synthesis and study of alkynyl surrogates of sterols (see Figure 15B), and their derived electrophiles, have occurred more recently, but the sterol compounds have a utility comparable to those in PUFA series.

Figure 15C shows an SDS gel for the lipid-adducted protein products that are formed from the reaction of human serum albumin (HSA) with alkynyl-secosterol A (*a*-seco A) at concentrations from 5 to 100 μM. After borohydride reduction to fix any adducts, Sharpless–Huisgen cycloaddition (click reaction) was carried out on the protein extracts, with the azide (shown in the figure) having an ethylene glycol linked to biotin. Gel electrophoresis of the protein product (western blotting using the streptavidin-AlexaFluor 680 conjugate) showed that *a*-seco A forms adducts with HSA, with protein aggregates being one consequence of adduction. The nature of the protein aggregates has not been established, but it is worth noting that the secosterol A and B dehydration product (secosterol – 2 H_2_O) could serve as a protein crosslinking agent, since it is both a Michael acceptor and an imine precursor.

Figure 15D shows a western blot comparison of the proteome modification by alkynyl sterols obtained from protein extracts of Neuro2a cells that were treated with 20 μM of either cholesterol (as a control), *a*-Chol, *a*-7-DHC, or *a*-DHCEp for 24 h. Proteins adducted with alkynyl lipids were ligated with biotin via a click reaction and adduction was determined as in Figure 15C. As shown in Figure 15D, *a*-Chol gives only background levels of adduction, with a blot intensity comparable to the cholesterol control. In contrast, both *a*-7-DHC and *a*-DHCEp show substantial levels of protein adduction covering a range of protein molecular weights. Since *a*-DHCEp is a reactive electrophile, the observation of significant protein adduction with this oxysterol is not surprising, but *a*-7-DHC is not itself an electrophile. The results obtained with this sterol imply that a significant conversion of *a*-7-DHC to alkynyl electrophilic species occurs over the course of the exposure, generating protein adducts in situ. This conclusion is consistent with the fact that 7-DHC is extremely vulnerable to free radical peroxidation, with electrophiles like 7-DHCEp being formed in the process.

Miyamoto and collaborators recently reported that Cu, Zn superoxide dismutase (SOD1) formed high molecular weight aggregates when the apo-enzyme was exposed to either seco A or seco B [155]. This observation is of interest since the accumulation of SOD1 aggregates has been associated with the development of familial amyotrophic lateral sclerosis ALS [12,156]. MALDI-TOF MS analysis showed that seco A and seco B react at multiple lysine sites on SOD1 with as many as five secosterols attached to the protein. The application of click methods similar to those described above for cytochrome c revealed that SOD1-secosterol adducts were primarily associated with high molecular weight aggregates. Therefore, click ligation of the secosterol-SOD1 product mixtures to a fluorophore and an SDS gel showed that the highest level of protein adduction was in the high molecular weight region of the gel. The protein adduction of highly hydrophobic sterol electrophiles will affect the protein structure, and it was suggested that protein aggregation is initiated by hydrophobic-hydrophobic interactions of sterol adducts. When SOD1 was exposed to *a*-4-HNE, dimers, trimers, and multimers were formed, but there was no evidence of very high molecular weight aggregates from this less hydrophobic electrophile.

**Figure 16.**
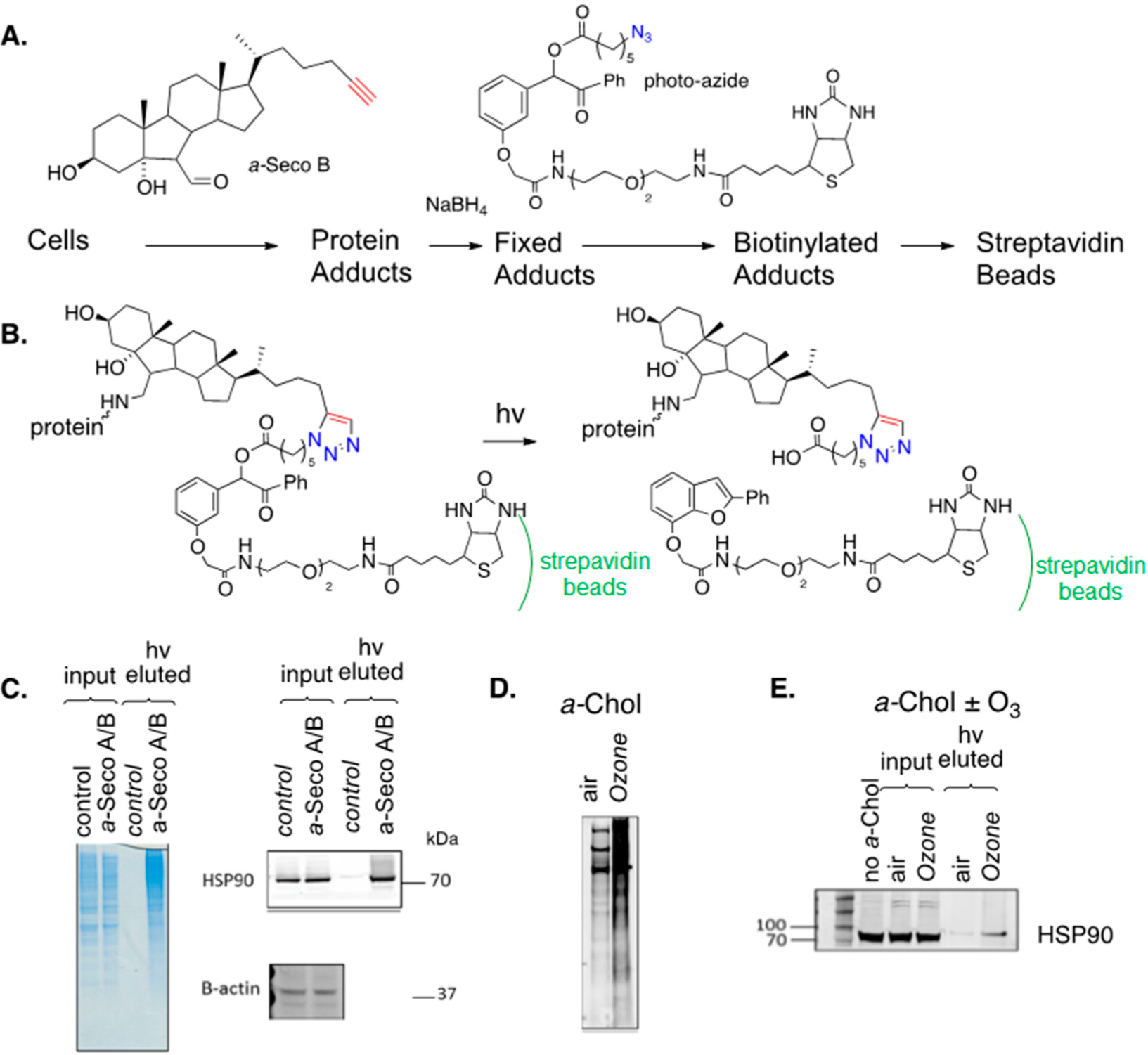
(**A**) Work-flow for the treatment of epithelial cells with *a*-seco A. (**B**) *a*-seco A adducted proteins were pulled-down on streptavidin beads. Beads were washed and adducted proteins photo-released (hv eluted). (**C**) SDS gels of control and *a*-seco A-treated cells were input to streptavidin beads and photo-released from the beads. Total adducted proteins are shown in blue, and HSP90 adducted proteins are presented in black and white. (**D**) Epithelial cells treated with *a*-Chol under air or ozone. Total protein adduct detected with anti-biotin fluorophore. (**E**) Pull-down on streptavidin beads and photo-release shows HSP90 adduction with *a*-Chol under air or ozone.

Speen et al. recently reported on the use of alkynyl sterols and secosterols to study protein adduction in human epithelial cells [157]. Cultured cells were exposed to alkynyl seco A or B and, after reduction with sodium borohydride, cellular proteins were treated with a biotin azide (photo-azide), as shown in Figure 16A. The specially designed photo-azide had a photo-cleavable linker insert between the azide and biotin functional groups so that the “catch and photo-release” sequence shown in Figure 16B could be applied. In the experiment, the mixture of un-modified proteins and biotinylated adducts was treated with a slurry of streptavidin beads, binding the adducted proteins to the beads and, after the unmodified proteins were removed from the beads by filtration, the protein adducts were released by photolysis and eluted from the beads. SDS gels of the protein input to the streptavidin beads and the photo-released (eluted) protein adducts are shown for the treated (exposure) and control cells in Figure 16C. The blue gels on the left of the figure show that photolysis of the beads released adducts with a range of molecular weights in the eluted/exposure lane. Adducts of specific proteins can be identified if selective antibodies for a protein are available, as shown in Figure 16C for the chaperone protein HSP90, which is an important therapeutic target for the treatment of a variety of cancers dependent on the chaperone-mediated stabilization of oncogenic proteins [158,159]. Levels of this protein input to the streptavidin beads were comparable for the control and treated cells, but no HSP90 was found in the photo-release fraction from the control cells, while this protein was evident in the release fraction of the treated cells, confirming that HSP90 is adducted by seco A/B in epithelial cells. In the same way, the liver X receptors LXRα and LXRβ were identified as targets for seco A/B adduction, as was the peroxisome proliferator-activated receptor PPAR.

Figure 16D,E show the results of an experiment in which *a*-Chol was incorporated into epithelial cells, followed by exposure of those cells to ozone. This experiment parallels the seco A/B study described above, but in this case, the electrophiles that formed adducts were generated in situ. Overall protein adduction from cellular treatment with *a*-Chol and ozone is shown in Figure 16D and the adduction of HSP90 by this same combination treatment is demonstrated in Figure 16E.

**Figure 17.**
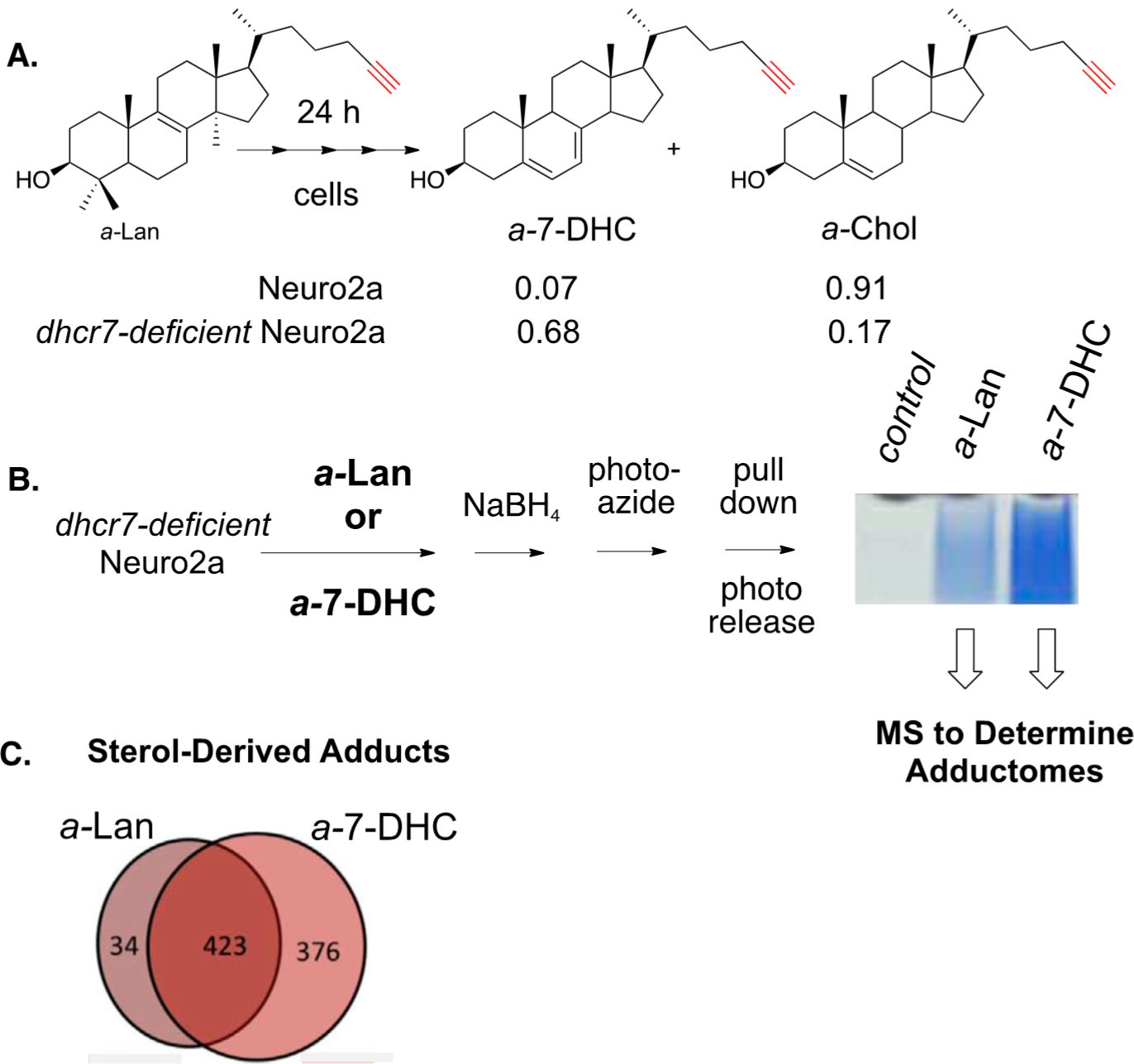
(**A**) Alkynyl sterols are viable surrogates for endogenous sterols in cell culture. Alkynyl lanosterol (*a*-Lan) undergoes multiple biosynthetic steps to give *a*-Chol in Neuro2a. In *dhcr7*-deficient Neuro2a, the biosynthesis is terminated at *a*-7-DHC. (**B**) *dhcr7*-deficient Neuro2a cells were treated with either *a*-Lan or *a*-7-DHC. Adducted proteins were pulled-down on streptavidin beads. Beads were washed and adducted proteins photo-released (hv eluted). SDS gels of control, *a*-Lan-treated, and *a*-7-DHC-treated cells were input to streptavidin beads and photo-released from the beads. Total adducted proteins, shown in blue, were subjected to proteomics assays. (**C**) *a*-7-DHC-derived electrophiles adducted nearly 800 proteins, whilst *a*-Lan-derived electrophiles adducted only 457 proteins, and 423 proteins were common to adduction by both *a*-Lan- and *a*-7-DHC-derived electrophiles.

The photo-azide strategy for adduct pull-down and photo-release was also used to define the adductome for 7-DHC-derived electrophiles in Neuro2a cells (see Figure 17) [160]. In this study, Neuro2a and *dhcr7*-deficient Neuro2a cells were incubated with alkynyl lanosterol (*a*-Lan) for 24 h and the alkynyl sterols present in the cells were assayed by HPLC-MS. In Neuro2a cells, most of the *a*-Lan was converted into *a*-Chol, demonstrating that the biosynthetic apparatus tolerated the alkynyl modification in the tail of the sterol. The same experiment carried out in *dhcr7*-deficient Neuro2a cells gave *a*-7-DHC as the major product, since the critical enzyme that carries out the last step in cholesterol biosynthesis is missing in these cells.

## 9. Questions and Prospects

HPLC-MS has been particularly helpful in defining mechanistic pathways and providing product profiles for the oxidation of cholesterol, 7-DHC, and other sterols. Product mixtures from radical chain processes are particularly complex, with recent reports expanding the sets of known electrophilic compounds. Cellular protein adduction by specific sterol-derived electrophiles has also been established, as have methods to identify the adductomes of various sterol-derived electrophiles. Indeed, oxysterol protein adduction appears to be a common outcome of many cellular oxidative exposures. Therefore, pieces of the puzzle linking oxidative stress exposure with electrophile formation and protein adduction are in place, but the picture remains blurry. In the simplest example, it was established that cholesterol reacts with ozone to yield electrophilic secosterols and that cellular exposure to secosterols gives protein adducts. The evidence that cellular ozone exposures leads to secosterol protein adducts has not yet been confirmed by a proteomics analysis from in vivo exposures. Radical chain oxidation provides an even more circumstantial picture, with the nature of the active electrophilic species in doubt for the radical chain-promoted oxidation of both cholesterol and 7-DHC.

In spite of the lack of detail outlined above, it seems highly likely that sterol-protein adduct formation occurs, raising general questions about the consequences of and control mechanisms for the process. The inhibition of oxidation serves as a control mechanism against adduct formation, with natural antioxidants forming a primary line of defense. If no electrophiles are generated, no adducts will be formed.

The disposal of protein adducts once formed would also appear to be a plausible defense mechanism. A 300 *m*/*z* hydrophobic sterol mass decorating any protein would be a significant structural perturbation and it seems likely that mechanisms exist to repair adducts and recover the native protein. In this regard, it seems worth mentioning that protein adduction should be reversible for many electrophiles. Secosterol adduction occurs by initial imine formation, which is a process that is chemically reversible. The reversible nature of adduct formation opens the possibility of an equilibrium distribution of a given secosterol among a set of available proteins in the locus of electrophile generation. Given this dynamic, it seems reasonable to speculate that a mechanism exists for the equilibration of a hydrophobic adduct from protein to protein, until a sink is found for disposal. We note that this idea is speculative and while it is conceptually pleasing, no evidence to support this suggestion has, to our knowledge, been presented.

Reversible electrophile-protein adduction, which can be considered a type of post-translational modification, can also serve as a signaling mechanism, because many of the protein targets of lipid electrophiles are involved in stress and inflammatory responses, such as Keap1/Nrf2, HSF1, PPARγ, and NF-κB [161]. Therefore, a small amount of electrophilic adduction likely serves as a protective mechanism in response to elevated oxidative stress. However, it remains to be elucidated whether sterol-derived electrophiles can also play the same roles in inducing protective cellular responses.

Given the recent recognition that the accumulation of phospholipid hydroperoxides drives the oxidative cell death modality now known as ferroptosis, it is compelling to suggest that sterol oxidation may contribute to either the initiation or execution of this process. As far as we are aware, all attention to date has been focused on (phospho)lipids. However, given the abundance of cholesterol and the integral structural role it plays in the lipid bilayers that are compromised during ferroptotic cell death, sterol oxidation and the products derived therefrom may be (the) key players. Along these lines, Birsoy and co-workers recently reported that cells devoid of squalene monooxygenase activity and that accumulate squalene at the expense of cholesterol are resistant to ferroptosis [162].

Many questions remain, but research on sterol peroxidation and sterol-derived electrophiles has advanced rapidly in recent years, with many tools now available to allow progress in the field. It seems likely that the links between oxidative stress, oxidizable sterols, oxysterol electrophiles, and the lipid-protein adductome will provide a fertile ground for exploration for years to come.
